# Epidemiology of pertussis in two Ibero-American countries with different vaccination policies: lessons derived from different surveillance systems

**DOI:** 10.1186/s12889-016-3844-9

**Published:** 2016-11-22

**Authors:** Rubén Solano, Josefa Masa-Calles, Zacarías Garib, Patricia Grullón, Sandy L. Santiago, Altagracia Brache, Ángela Domínguez, Joan A. Caylà

**Affiliations:** 1 CIBER Epidemiology and Public Health (CIBERESP), Epidemiology Service-Barcelona Public Health Agency, Pl. Lesseps 1, 08023 Barcelona, Spain; 2 National Centre for Epidemiology, Carlos III Health Institute, Madrid, Spain; 3 Extended Immunizations Programme, Dominican Ministry of Health and Social Assistance, Santo Domingo, Dominican Republic; 4 Department of Public Health, University of Barcelona, Barcelona, Spain; 5 Epidemiology Service, Barcelona Public Health Agency, Barcelona, Spain

**Keywords:** *Bordetella pertussis*, Whooping cough, Infectious disease epidemiology, Surveillance, Spain, Dominican Republic

## Abstract

**Background:**

Pertussis is a re-emerging disease worldwide despite its high vaccination coverage. European and Latin-American countries have used different surveillance and vaccination policies against pertussis. We compared the epidemiology of this disease in two Ibero-American countries with different vaccination and surveillance policies.

**Methods:**

We compared the epidemiology of pertussis in Spain and the Dominican Republic (DR). We present a 10-year observational study of reported pertussis based on suspected and/or probable cases of pertussis identified by the national mandatory reporting system in both countries between 2005 and 2014. Both countries have a similar case definition for pertussis surveillance, although Spain applies laboratory testing, and uses real time PCR and/or culture for case confirmation while in DR only probable and/or suspected cases are reported. We analyzed incidence, hospitalization, case-fatality rates, mortality and vaccination coverage.

**Results:**

The average annual incidence in children aged <1 year was 3.40/100,000 population in Spain and 12.15/100,000 in DR (*p* = 0.01). While the incidence in DR was generally higher than in Spain, in 2011 it was six times higher in Spain than in DR. The highest infant mortality in Spain was 0.017/100,000 in 2011, and the highest in DR was 0.08/100,000 in 2014 (*p* = 0.01). The proportion of hospitalized cases per year among children <1 year varied between 22.0% and 93.7% in Spain, and between 1.1% and 29.4% in DR (*p* = 0.0002), while mortality varied from 0 to 0.017 and 0 to 0.08 per 100,000 population in Spain and DR, respectively (*p* = 0.001). Vaccination coverage was 96.5% in Spain and 82.2% in DR (*p* = 0.001).

**Conclusions:**

Pertussis is a public health problem in both countries. Surveillance, prevention and control measures should be improved, especially in DR. Current vaccination programs are not sufficient for preventing continued pertussis transmission, even in Spain which has high vaccination coverage.

## Background

Approaches to surveillance, prevention (including immunization) and control of pertussis vary markedly worldwide, even between European countries. For good epidemiological control of mandatory notifiable diseases such as pertussis, it is important to limit under-reporting, and to have alternative surveillance systems in place (microbiology results, hospital discharges, etc.). The current incidence of this disease seems to be influenced by vaccination coverage, since most countries with extensive pertussis vaccine coverage in infants have achieved a significant reduction in morbidity and mortality in this population [1, 2]. However, several studies have reported differences in pertussis incidence in countries with different vaccination programs [1, 3, 4]. Laboratory surveillance is essential for guiding vaccination policy, but this surveillance is lacking in many Latin-American countries. The general lack of specific laboratory diagnosis by real time polymerase chain reaction (PCR) and serology in Latin America is a major technology gap in the region’s ability to effectively control pertussis [5].

Reports from several regions in Spain and in some Latin-American countries highlight increased incidence, hospitalization and mortality due to pertussis infection, especially among infants who are too young to be immunized [6–9].

European and Latin-American countries have used different vaccination strategies against pertussis (Table 1) [10]. In most European Union countries, whole-cell pertussis vaccines have recently been replaced with acellular vaccine combinations containing diphtheria, tetanus toxoids and pertussis components (DTaP and Tdap). However, pertussis remains endemic despite high coverage with whole-cell (DTwP) or acellular (DTaP) vaccines. This is likely due to decreasing immunity, and demands new vaccination strategies that provide long-lasting immunity [11]. Hegerle N *et al*. reported in 2014 that some new strains of pertussis show a loss in production of pertactin (*prn*), a virulence factor included in various acellular pertussis vaccines against both *B. pertussis* and the closely related *B. parapertussis*. This phenomenon highlights the need to reassess the efficacy and effectiveness of these vaccines [12].

**Table 1:** Incidence of notified pertussis, vaccines, vaccination schedules and coverage in Spain and Dominican Republic, 2005–2014

Country	Global Incidence per 100,000 inhab.^a^	Incidence per 100,000 inhab. ≤1 year-old	Vaccine	Manufacturer	Global coverage^b^ (%)	Vaccination Schedule^c^ (type vaccine)
Spain (Iberian country)	3.4 (0.5–8,4)	813.3	*Infanrix Hexa*® *Infanrix VPI Hib*® *Pentavac*®	GlaxoSmithKline Biologicals, S.A. Sanofi Pasteur MSD, S.A.	96.5	Primary: 2, 4, 6 mo. Booster: 15–18 mo and 4–6 yr (DTaP)
DR (Caribbean country)	12.1 (1.0–37.0)	132.0	*Quinvaxem*® *Tritanrix*® *HB* *DTCHepB.Hib*®	GlaxoSmithKline Biologicals, S.A.	82.2	Primary: 2, 4, 6 mo. Booster: 18 mo and 4 yr (DTwP)

^a^Care should be taken when comparing incidences between countries because of the different surveillance and diagnostic methods used

^b^coverage in children aged 2, 4 and 6 months (primary series coverage in children)

^c^two booster doses are given in DR and Spain at the age of 18 months and 4 years-old, and between 4–6 years

The aim of this study was to compare the epidemiology of pertussis in two geographically distant countries, Spain and the Dominican Republic (DR), which have different vaccination and surveillance policies.

## Methods

### Data sources

Reports of clinically suspected and laboratory confirmed cases of pertussis in Spain and DR during 2005-2014 were obtained from the Spanish Epidemiological Surveillance Network at the National Centre for Epidemiology, Carlos III Health Institute (RENAVE-CNE-ISCIII), and the Dominican Ministry of Health and Social Assistance Expanded Program on Immunization (SESPAS-PAI), respectively. Additional information regarding vaccination status was recovered from epidemiological records, where available. Data on pertussis-related hospital admissions and deaths were obtained from the Spanish national minimum basic data set (MBDS) and from the Institutes of Statistics in both countries, respectively [13–15].

Hospitalization data were obtained from the Ministry of Health, Social Services and Equality in Spain, and from the Ministry of Health in DR. We used demographic data from the National Statistics Office of each country to calculate rates of incidence, hospitalization and mortality between 2005 and 2014, and for each sex and age group [16]. Because of a lack of laboratory confirmation, it was not possible to assess progression of confirmed cases in DR.

#### Definitions

In Spain, the clinical case definition for pertussis was that established by RENAVE-CNE-ISCIII: until 2012 it was “a cough lasting ≥2 weeks, followed by paroxysmal cough, inspiratory whoop, post-tussive vomiting in absence of other apparent cause”. Since 2013 apnea in infants younger than 1 year of year was added as clinical criteria. Laboratory case confirmation was performed on nasopharyngeal secretion samples using culture (Bordet-Gengou and/or Regan-Lowe) and/or PCR for nucleic acid detection of *B. pertussis* in clinical samples. Suspected cases were required to meet the clinical case definition established by RENAVE-CNE-ISCIII. Until 2011, probable cases were those that met the clinical case definition, were not laboratory confirmed and were not epidemiologically linked to a laboratory confirmed case. In 2013, probable cases were considered those that met the clinical case definition and were epidemiologically linked with a laboratory confirmed case [17, 18].

In DR, positive cases of pertussis were diagnosed according to the World Health Organization’s clinical case definition: “a cough lasting ≥2 weeks with at least one of the following symptoms: paroxysms of coughing, inspiratory whooping or post-tussive vomiting or apnea without other apparent causes”, and cases were not laboratory-confirmed [19].

The cause of hospitalization was considered to be pertussis in cases where the main diagnosis was coded as 033.0/033.1/033.8/033.9 according to the International Classification of Diseases, Ninth Revision, Clinical Modification (ICD-9-CM) [20].

### Immunization scheme and coverage

Vaccinations included in the childhood immunization schedule in Spain are financed by the National Health System (SNS), and are performed according to the recommendations of the Spanish Inter-territorial Council. These recommendations take into account epidemiological data, as well as evidence regarding the safety, effectiveness and efficiency of current vaccines. During the study period, Spanish children received either DTaP (Infanrix hexa®) at the age of 2, 4, and 6 months. The Spanish SNS switched to using acellular vaccines in 2005, and the currently recommended DTaP vaccination schedule at a national level consists of DTaP at the age of 2, 4, 6, and 18 months, followed by a reminder dose between the age of 4 and 6 years [21].

The immunization schedule for Dominican children aged 2 months to 6 years includes (Quinvaxem®) and (Tritanrix-HB + HIBERIX®) vaccines, both including DTwP. In DR the DTwP vaccine is recommended at 2, 4, 6 and 18 months, and 4 years of age, and is financed by the Pan American Health Organization (PAHO) [20]. Cases who had received all vaccination doses corresponding to their age were classified as adequately vaccinated. Cases who had received fewer than the appropriate number of doses for their age were classified as inadequately vaccinated. Cases who had received no vaccination at all were classified as unvaccinated. Vaccination status in both countries was ascertained from the patient’s vaccination card, or from the corresponding surveillance system (updated by the visiting physician based on an oral report by the child’s mother).

### Statistical analysis

We performed a descriptive analysis of the patients’ clinical characteristics, and of the presenting illness and its progression over time. For this analysis, we searched for association between key characteristics of pertussis and mortality.

The following age groups were considered for the analysis: <1, 1-4, 5-9, 10-14 and ≥15 years of age.

Statistical significance of observed associations was evaluated using the Chi-square or Fisher’s exact test for categorical variables, and the Wilcoxon rank-sum test for continuous variables. Statistical significance was set at *p* < 0.05 for all analyses. All statistical analyses were performed using the Statistical Package for Social Sciences (SPSS® version 18.0, Chicago, IL, USA) for Windows (Microsoft Corp., Redmond, WA, USA).

## Results

### Number of cases, incidence, hospitalizations and deaths

In Spain, there were 4,578, 3,147, 2,392 and 2,153 reported cases of pertussis among children aged <1, 1-4, 5-9, and 10–14 years, respectively, and 3,470 among adolescents and adults aged ≥15 years. The number of laboratory-confirmed cases in these age groups was 54.6% (2502/4,578), 37.0% (1163/3147), 46.9% (1122/2392), 47.4% (1020/2153) and 40.2% (1394/3470), respectively, and the number of probable cases was 45.3% (2076/4,578), 63.1% (1984/3147), 53.1% (1270/2392), 52.6% (1133/2153), respectively.

In DR, 260 cases were reported among infants <1 year, and only 4 cases were reported in children aged 1-4 years. No cases were reported in the other age groups. All cases were categorized as suspected or probable cases (median 1.0).

**Fig. 1:**
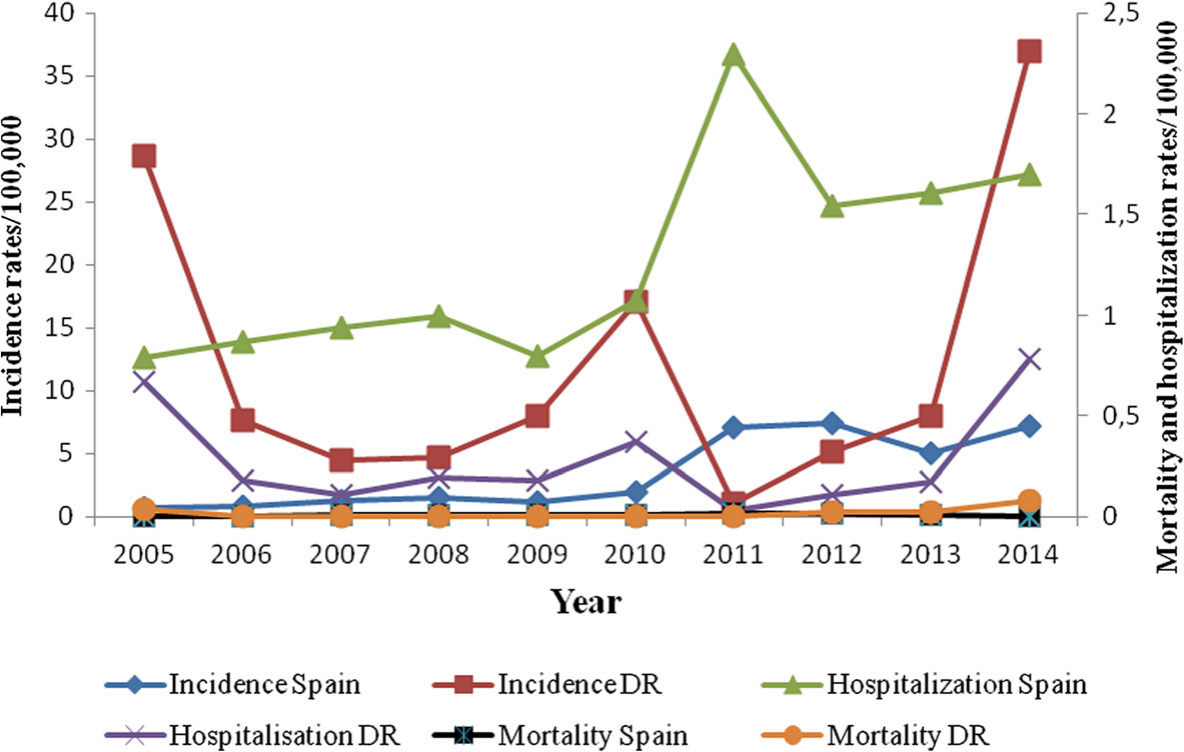
Age-standarized incidence, and rates of hospitalization and mortality due to pertussis in Spain and the Dominican Republic, 2005–2014

The overall incidence of pertussis in DR was higher than in Spain, especially in 2014, although there was an isolated inversion of this trend in 2011, when the incidence in Spain was six times that in DR (Fig. 1). The specific peak of incidence in DR underwent an upward trend in between 2012 to 2014.

The highest differences between the two countries in terms of the epidemiological indicators studies were in the 2014 incidence (37.0/100,000 in DR and 7.18/100,000 in Spain), the 2014 hospitalization rate (0.78/100,000 inhabitants in DR and 1.7/100,000 in Spain), and the 2014 mortality (0.08/100,000 in DR and 0.001/100,000 inhabitants in Spain).

As of 2010, the number of hospitalizations due to pertussis increased in Spain, peaking in 2011 with 2.3 hospitalizations per 100,000 inhabitants. The trend in age-standardized hospitalizations was similar to that for case reports in both countries (Fig. 1); however, the rates varied throughout the study period. In Spain, the percentage of hospitalized cases was very high during the initial years of the study (2005–2006; 88.6%–93.7%), but was only 20–40% between 2011 and 2014. In DR, 23.3% and 29.4% of cases were hospitalized in 2005 and 2014, respectively.

**Fig. 2:**
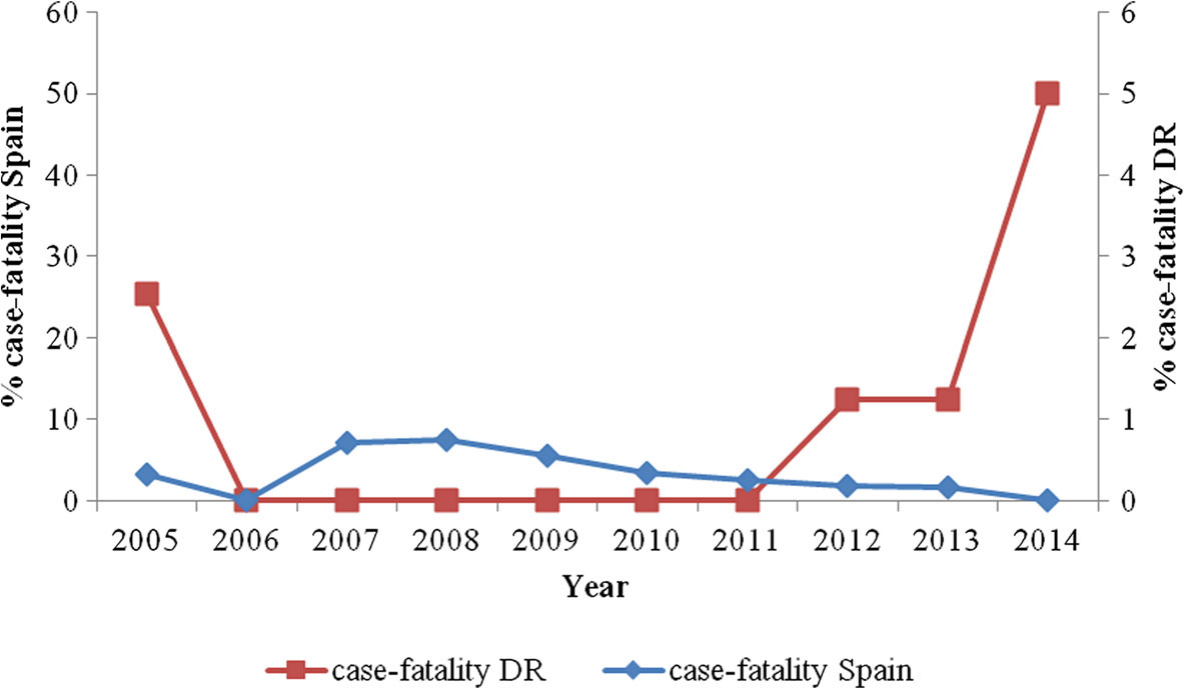
Evolution of case-fatality rates of pertussis in Spain and the Dominican Republic, 2005–2014

The rate of mortality due to pertussis in Spain was 0.01, 0.02 and 0.01 per 100,000 inhabitants in 2008, 2011 and 2012, respectively. All deaths due to pertussis in both countries occurred in young infants who were incompletely immunized or unvaccinated. In DR, data on the number of deaths caused by pertussis were only available for 2005 and 2012–2014, and were limited to the <1 year age group (Fig. 1). Pertussis mortality increased significantly from 0.04 to 0.08 per 100,000 inhabitants between 2005 and 2014 (*p* = 0.01). Also, the case-fatality rate was higher in DR, reaching 25.4% and 50.0% in 2005 and 2014, respectively; conversely, in Spain, it tended to stay below 5% (Fig. 2).

### Vaccination status

Throughout the study period, the overall rate of vaccination coverage was 96.5% in Spain and 82.2% in DR (*p* = 0.001) (Table 1). Coverage in DR varied significantly between regions, ranging from 66.1% to 88.0%.

**Fig. 3:**
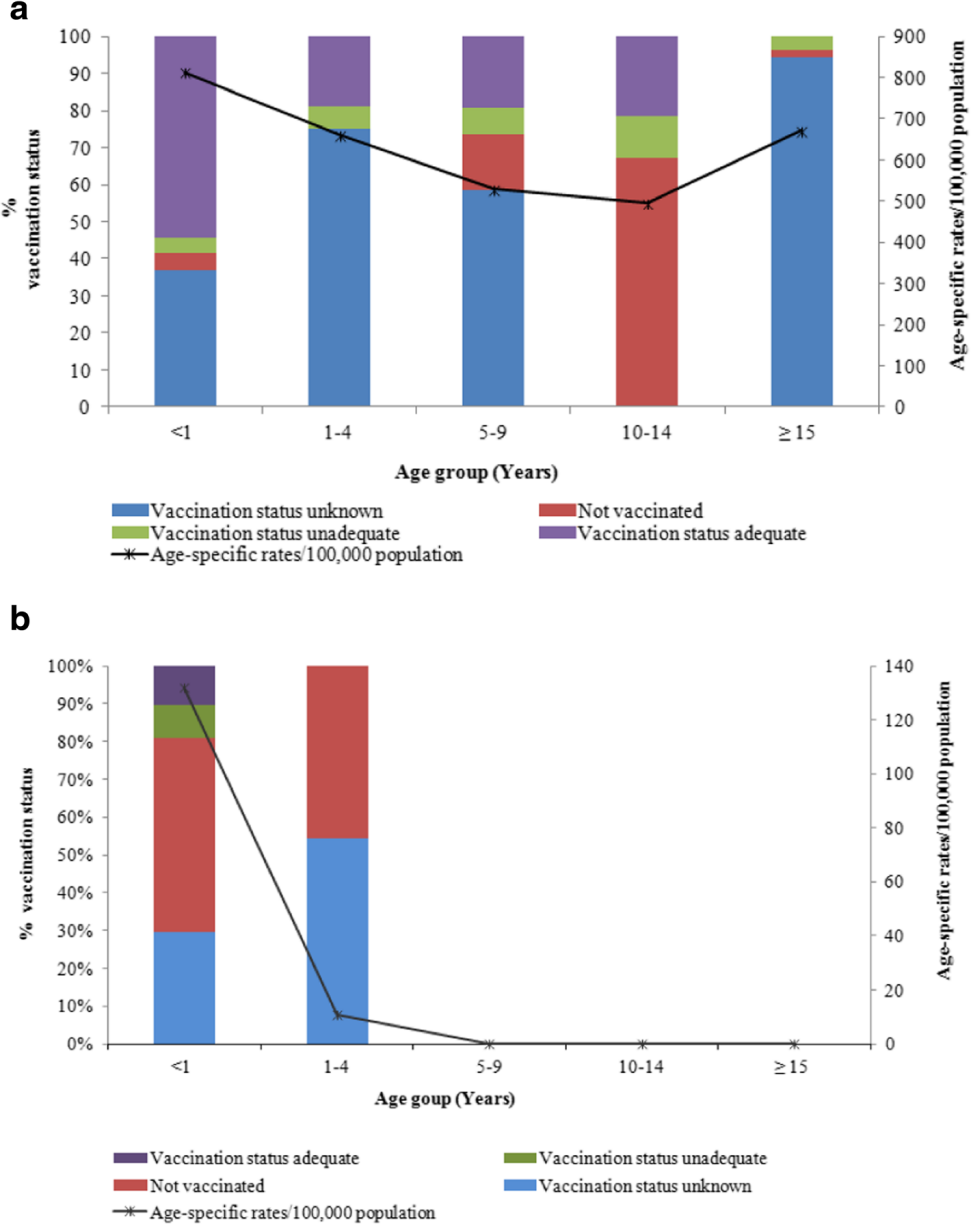
**a** Reported cases of pertussis according to vaccination status, and incidence rate, stratified by age group. Spain, 2005–2014. **b** Reported cases of pertussis according to vaccination status, and incidence rates, stratified by age groups. Dominican Republic, 2005–2014

The profiles of vaccinated children and those with unknown vaccination status differed between countries; 54.2% of cases aged <1 year in Spain had an adequate vaccination status, while in DR this rate was 10.3%. The percentage of unvaccinated children <1 year of age was higher in DR (51.0%) than in Spain (4.4%) (*p* = 0.001); among 1- to 4-year-olds this value was 45.5% and 0%, respectively. The proportion of children with pertussis diagnosis and vaccination status in both countries, are show in Fig. 3a and b. Furthermore, among notified cases with information on immunization status, 19.4% of children aged 5–9 had receive four doses of vaccine in Spain. Therefore, the highest proportion of adequately vaccinated patients was in children <1 year with a high incidence rate of 813.3/100,000 inhabitants. The ≥15 years age group had the highest percentage of cases with unknown immunization status (94.4%). In DR, most of the information collected regarding the vaccination status of children refers to the first series of DTwP vaccinations, because most reported cases (95.1%) were children aged <1 year.

In Spain, 31.4% of children aged 1–4 had unknown immunization status. In the <1 year age group, the proportion of cases with unknown immunization status was low in both Spain and DR (19.0% and 31.4%, respectively).

## Discussion

In this study carried out in Spain and DR, we observed important differences in pertussis epidemiology: higher incidence, and case-fatality and mortality rates in DR, but higher rates of hospitalization and vaccination coverage in Spain.

These data show that pertussis is still a public health problem both in Spain and DR. Also, surveillance data in DR support the need for improvement because of the potential importance of under-reporting (low incidence and vaccination coverage, combined with a high case fatality rate) [22]. Furthermore, none of the cases in DR were laboratory-confirmed, but rather were diagnosed as pertussis by physicians on the basis of clinical symptoms. These deficiencies in the surveillance of pertussis have also been reported in other Latin-American countries [22, 23].

Overall, most cases in these countries are diagnosed on the basis of bacterial culture, the sensitivity of which varies according to the stage of the infection [24, 25]. Adherence to screening recommendations and access to healthcare influence early detection of pertussis infection. However, in Spain these recommendations are not followed consistently. In this sense, routine pertussis screening has been performed since the 1990s during outbreaks in the USA in settings such as schools, day care centers, and hospitals (for symptomatic patients with suspected pertussis), which promotes prompt administration of antibiotics for high risk close contacts and suspected cases [26, 27].

### Incidence

Several aspects could have influenced the results of this study. For example, it is important to highlight under-reporting as a persistent problem in controlling infectious diseases, despite mandatory notification by physicians. We must also consider possible differences in the quality of historical data and overall management of surveillance, prevention and control of contacts, as noted in other studies [28–30].

Interestingly, epidemiological data show that pertussis incidence has increased in all age groups in Latin-America and Europe, where acellular pertussis vaccines are used, although an increase was also observed in some countries that use whole-cell vaccines, such as DR, where vaccine coverage is low, and implementing and improving laboratory diagnostic methods is crucial [31]. For this reason, some European countries have shifted from pertussis testing based on risk assessment to pertussis screening in certain households, schools and community settings. This screening consists of testing for infections in asymptomatic people, a protocol that is never used in DR and rarely in Spain [32–35].

### Hospitalization

Similarly, the percentage of hospitalized cases is conditioned by the quality of the monitoring and case reporting. The high rate of pertussis hospitalization in young infants in both countries considered in this study may be due to a tendency to only report serious cases to their respective surveillance systems.

Another factor that may have influenced our results is the lack of access to health-care insurance in DR, with a mortality rate of 0.3 deaths per 1,000 live births in 2008. The main causes were child malnutrition and communicable diseases (acute respiratory diseases), which are notably more common than in other Latin American and Caribbean countries with similar economic conditions [36].

The percentage of cases with confirmed diagnosis was highest in Spain, which has increasing hospitalization rates, and the majority (92%) of hospitalizations occurred in children under one year of age. However, between 2011 and 2014, hospitalization trends in Spain have begun to approach reality because of improved reporting and hospitalization of cases of pertussis [20]. There was also a significant increase in hospitalization rates during the study period in both countries due to more accurate and timely reporting of hospitalizations, which represent the most severe cases, as a result of using electronic disease-reporting systems [37, 38]. In the USA, a similar time trend was reported in children hospitalized with bronchiolitis during the months of November to March, between 2007 to 2010 [39].

### Mortality

The average infant mortality in Spain was ~3.1 deaths per 1,000 live births in 2012, which is below average for the European Union (4/1000 live births) [15]. The infant mortality rate in DR was ~21.3 per 1,000 live births in 2012, decreasing to ~19.6 per 1,000 live births in 2014. Previous studies have analyzed the main causes of death in children in Spain, namely cancer, heart and cerebrovascular diseases, and some infectious diseases, including pertussis [40, 41]. This confirms the need to modify vaccination strategies in this country to better serve the most vulnerable population groups [8]. The first should involve vaccinating pregnant women, which directly protects through the passive transfer of pertussis antibodies. The second strategy, cocooning, involves vaccinating parents, caregivers, and other close contacts, which indirectly protects infants from transmission by preventing disease in those in close proximity [42].

The overall mortality rate in DR rose from 5.99% in 2003 to 6.03% in 2012 and 6.05% in 2013, exacerbating the country’s mortality rate ranking in Latin-America. Insufficient coverage by the health system, underreporting of disease, lack of medical certification, and ill-defined certificates are some of the barriers to understanding and evaluating the current processes [14, 43].

### Vaccination

In 2007, pentavalent (DPT-HB-Hib) vaccination coverage (69.6%) among children aged <1 year was low in DR, a country where less than half the population has health insurance [14]. This may also be due to high rates of social disadvantage together with a lack of a network of social and family support. However, Latin-American countries have good capacity to conduct vaccination programs, and there is generous government support, in accordance with World Health Organization (WHO) recommendations issued since 1992 [44]. The Revolving Fund of the Pan American Health Organization (PAHO) contributed to this achievement by pushing forward policy changes that support national immunization programs, such as vaccine legislation and economic actions to ensure the delivery of services [45]. Whole-cell pertussis vaccines (DTwP), which appear to be quite effective, are used throughout Latin America. A 4-dose schedule is typically used (2, 4, 6 and 18 months), and a booster dose is given between the ages of 4 and 6; this treatment reaches >90% of the population in most regions, but is incomplete in regions with political or geographical barriers, resulting in poor uptake of combination vaccines and low coverage [9, 46]. The situation in Spain is different to that in DR, suggesting a progressive accumulation of susceptible individuals due to waning immunity following years of low incidence [6, 47–49].

In 2015, Spain adopted the recommendation to vaccinate pregnant women as an alternative immunization strategy to protect newborns, with the aim of protecting infants during the first weeks of life [19, 50].

The Advisory Committee on Vaccines of the Spanish Association of Paediatrics updated their immunization schedule and only reasserts its recommendation to include vaccination strategies, with dTap for pregnant women and household contacts of the newborn [51].

Currently, no data are available in both countries to know about levels of compliance with the gynaecologists' and public health services' recommendation to vaccinate pregnant women.

In Spain there is now an ongoing project to estimate the effectiveness of the vaccine in pregnant women.

A possible limitation of this work is the fact that it is a retrospective database study, which makes it difficult to capture all of the relevant information for the 10-year period studied. The use of SESPAS-PAI surveillance data for case detection in DR could result in under-estimation of infection rates. A further limitation was that suspected and probable reported cases in DR, these cases were subjectively assessed according to clinical criteria were not specifically of pertussis, such as lymphocytosis. Lack of comprehensive data from the surveillance system in DR results in several limitations, and the underestimation of pertussis in all age groups may be mainly related to its atypical clinical manifestations and the lack of laboratory confirmation (clinical and demographic survey data).

### Implications for public health policy

It is difficult to compare the epidemiology of pertussis in Spain and DR. The mandatory notification system in DR is not very effective, and laboratory confirmation is not done, making it impossible to determine the true epidemiological incidence in this country. The differences in historical health policies seem more determinative in this comparison, and there is a need to improve the vaccination coverage with effective vaccines. The difference in age patterns of reported cases between countries likely accounts for this contrasting observation. Despite the lack of comprehensive data in DR, this should not hamper the use of active prophylactic measures designed to limit the impact of waning immunity against pertussis. Most children were likely to have received three doses of DTaP vaccine in Spain and DTwP vaccine in DR within the first year of life. The profiles of vaccinated children and those with unknown vaccination status were different in each country.

## Conclusions

The observed differences between the two countries suggest that pertussis control programs should be improved by developing new active surveillance systems, vaccination strategies and diagnostic technologies, especially in DR. We provide lessons for the future evaluation of public health interventions. The results of several studies in Belgium and the UK support the recommendation to immunize during pregnancy, and provide additional evidence of possible interference by maternal antibodies to the fetus. This reduces incidence and mortality in children <1 year-old [52, 53], so it would be advisable to adopt this policy systematically in both Spain and DR.

## Abbreviations

DR: Dominican Republic
DTaP: Diphtheria-tetanus-acellular pertussis
DTwP: Diphtheria-tetanus-whole cell pertussis
ICD-9-CM: International classification of diseases, ninth revision, clinical modification
MBDS: Spanish national minimum basic data set
PAHO: Pan American Health Organization
PCR: Real time polymerase chain reaction
prn: Pertactin
RENAVE-CNE-ISCIII: Spanish epidemiological surveillance network at the national centre for epidemiology, Carlos III Health Institute
SESPAS-PAI: Dominican ministry of health and social assistance expanded program on immunization
SNS: Spanish national health system
WHO: World Health Organization
